# Glaucoma Classification Through SSVEP-Derived ON- and OFF-Pathway Features

**DOI:** 10.1167/tvst.15.1.2

**Published:** 2026-01-05

**Authors:** Martin T. W. Scott, Hui Xu, Alexandra Yakovleva, Robert Tibshirani, Jeffrey L. Goldberg, Anthony M. Norcia

**Affiliations:** 1 Department of Psychology, Stanford University, Stanford, CA, USA; 2 Department of Statistics, Stanford University, Stanford, CA, USA; 3 Department of Ophthalmology, Spencer Center for Vision Research, Byers Eye Institute, Stanford University, Stanford, CA, USA

**Keywords:** glaucoma, ON/OFF pathways, SSVEP, phase delay, machine learning classification

## Abstract

**Purpose:**

This work aims to evaluate the relative contribution of the amplitude and phase of both ON- and OFF-pathway biased steady-state visually evoked potentials (SSVEPs) to the classification of patients with glaucoma from healthy controls.

**Methods:**

SSVEPs were recorded for sawtooth luminance increments (ON-biasing) and decrements (OFF-biasing), modulating at a temporal frequency of 2.73 Hz. SSVEP data from 98 adults with glaucoma and 71 controls were used to train a set of logistic regressions. Data were partitioned prior to training to investigate the relative contribution to classification for amplitude and phase features derived from ON- versus OFF-pathway stimulation.

**Results:**

We report moderate overall classification accuracy (area under the curve ∼0.7). Classification based solely on signal phase features significantly outperformed classification based solely on signal amplitude features. Classification using OFF-pathway biasing features produced a statistically significant improvement in classification only when training on signal amplitude features. This OFF advantage was not conserved in a dataset with low signal-to-noise eyes removed.

**Conclusions:**

Our findings highlight the informational value of signal phase, a metric often omitted in applications of the SSVEP to glaucoma and other optic neuropathies. Additionally, our results suggest that OFF-pathway amplitude features may be less vulnerable to the limitations imposed by a low signal-to-noise ratio. However, they are not indicative of a gross difference in glaucoma classification performance between ON- and OFF-pathway biased features.

**Translational Relevance:**

Electrophysiological estimates of visual signal delay should be considered in future clinical diagnostic tools as they make a material contribution to the classification of glaucomatous eyes.

## Introduction

Multiple electrophysiological studies have performed individual patient classification using the isolated-check visually evoked potential (icVEP)—a form of steady-state visually evoked potential (SSVEP).^1^^–^^10^ The icVEP protocol has variations that are designed to bias neural responses (measured at the scalp) toward either the ON or OFF pathway. This biasing is accomplished by presenting a rectilinear grid of small patches that sinusoidally modulate their luminance either above the luminance of an otherwise uniform background (ON-pathway favoring) or below it (OFF-pathway favoring). Recent work using this paradigm in glaucoma has focused on ON-favoring stimuli. This choice was based on early group-level and individual-level analyses^1^^,^^11^ showing greater effects of glaucoma (and best classification performance) with ON-biasing sinusoidal stimulation. Using a simple icVEP signal-to-noise ratio (SNR) threshold, the neural response evoked by this stimulus can reportedly separate patients from controls with moderate to good success (receiver operating characteristic area under the curves [ROC-AUCs] of 0.74–0.94), approaching the glaucoma classification performance of other optical coherence tomography images^12^ and Humphrey visual field data.^13^

Recent work in mouse models of glaucoma has suggested that OFF retinal ganglion cells (RGCs) are more susceptible to damage than ON RGCs.^14^^–^^17^ In human patients with glaucoma, these reports have been corroborated by noninvasive electrophysiological measures of ON- and OFF-pathway function^18^^–^^21^ and also reflected in retinal imaging of the ON and OFF sublaminae of the inner plexiform layer.^22^ In each of these cases, evidence for preferential OFF-pathway damage was found using group-level statistical analyses. While group-level analysis is a valuable research strategy for directing further exploration of biomarkers for glaucoma, establishing individual patient metrics for glaucoma detection is important as well. Given recent SSVEP and electroretinogram group-level results suggesting greater OFF-pathway loss in glaucoma, here we revisit the relative accuracy of binary classification (glaucoma patient versus control) of individuals on the basis of ON- and OFF-pathway biasing SSVEPs in a large sample.

Our approach differs from previous classification-based SSVEP studies in a few ways. First, we use sawtooth modulation instead of sinusoidal modulation (as in Norcia et al.^18^), which has the potential to more effectively bias neural responses toward either the ON or OFF pathways.^23^ Second, we recorded 128-channel electroencephalography (EEG) rather than single-channel EEG and used spatial filtering for dimension reduction and signal enhancement. Spatial filtering was accomplished via an objective dimension reduction approach that weights EEG channels based on a fundamental aspect of the SSVEP: channels with stimulus-locked responses are the channels of interest.^24^ Third, whereas previous work focuses on the SNR of a single response harmonic, we retain signal amplitude and the phase at several response harmonics for use in multifeature binary classification. This is based on macaque^25^ and human^10^^,^^18^^,^^26^^–^^30^ evidence that glaucoma affects the signal phase, as well as reports of glaucoma producing demyelination of the optic nerve.^31^^,^^32^ Notably, our inclusion of multiple harmonics is permitted by our use of slower stimulus modulation frequencies. Fourth, we use a cross-validation method that allows for the inclusion of both participants’ eyes in the training data (where available) and reduces the likelihood of overfitting. Our results highlight the overall importance of considering both amplitude and phase information and suggest that OFF-pathway features may be more robust to low SNR.

## Methods

### Participants

Experiments proceeded after approval by the Institutional Review Board of Stanford University. Written informed consent was obtained from all participants, and all research conformed to the tenets of the Declaration of Helsinki for the use of human participants. The participants were recruited from the Stanford University community or were patients at glaucoma/optometry clinics of the Byers Eye Institute at Stanford University. Participants included 98 adults with glaucoma (mean age = 59.3; 39 females, 54 males, 5 unknown) and 71 adults without glaucoma or other ocular pathology (mean age = 61.7; 35 females, 29 males, 7 unknown). Data from 177 eyes of 98 patients and 140 eyes of 71 controls were used in the classification analysis.

**Figure 1.**
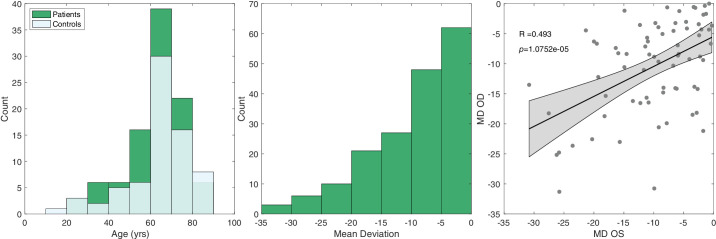
Demographic information of participants. *Left*: Distribution of ages for patients and controls. *Middle*: Distribution of MD values for patients. *Right*: Left-eye (OS) MD scores plotted against the right-eye (OD) MD scores and the fit of a linear regression (shaded region = 95% CI on the fit).

Inclusion criteria for patients with glaucoma included a diagnosis of glaucoma, best-corrected visual acuity of 20/70 or better in the study eye(s), absence of other ophthalmic conditions that may impact vision, and cognitive abilities sufficient to participate in the study. Glaucoma was diagnosed based on glaucomatous optic nerve head damage on fundoscopy and retinal nerve fiber layer thinning on optical coherence tomography (respectively) and typical visual field loss on the 24-2 Humphrey visual field. Typical field loss was defined as a positive glaucoma hemifield test or a cluster of at least three points below *P* = 0.05, with at least one point below *P* = 0.01. Humphrey visual field testing was performed for all patients with glaucoma on the day of or within 3 months prior to the visually evoked potential (VEP) recording. Group-level data from 61 of our patients and 37 control participants have been reported previously.^18^ The distributions of mean deviations (MDs) and ages of the patient sample are shown in Figure 1. For MD correlation (Fig. 1, right), 72 patients with both Oculus Dexter & Oculus Sinister measurements were included, and a regression line was used in the “fitlm()” function in MATLAB 2022b.

### Visual Stimuli

**Figure 2.**
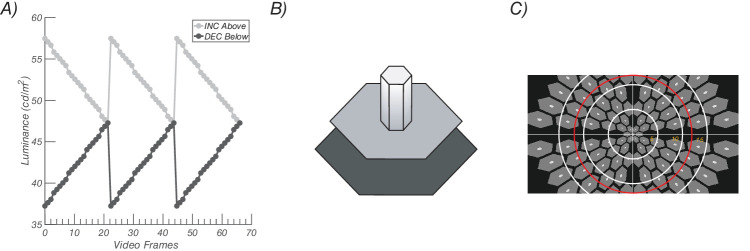
Visual stimulation paradigm. (**A**) Sawtooth stimulation profiles for probes, with incremental probes presented above the pedestal luminance and decremental probes below. (**B**) Probe-on-pedestal display element. The sawtooth-modulated probes (small white element rising from the gray pedestal) were sawtooth modulated, as shown here above the pedestal. The probe was 20% of the size of the pedestal. The pedestal was surrounded by a larger *black background*. (**C**) Scaled stimulus array. *White rings* indicate 5° increments of eccentricity from central fixation. The *red ring* is 12° in radius.

Responses to ON- and OFF-pathway biasing stimuli were measured using a hexagonal array of flickering probes. The entire array subtended 42° × 25° of visual angle. Two types of hexagonal elements are present in the stimulus array: probes and pedestals. Pedestal hexagons are larger elements that have a fixed luminance of 47 cd/m^2^, while probe hexagons are smaller elements within pedestals with luminance modulation that was experimentally manipulated. All hexagons were presented against a low-luminance background of 11 cd/m^2^ (see Fig. 2B). Elements that straddled the horizontal and vertical meridian were eliminated (see Fig. 2C for a schematic example). Probe elements were temporally modulated according to a sawtooth profile, the fast phase of which was set to bias evoked responses either toward the ON or OFF pathway.^23^ ON-pathway biasing stimuli were defined as probes that rapidly increased in luminance and slowly decreased, while OFF-pathway biasing stimuli were defined as probes that rapidly decreased in luminance and slowly increased (see waveforms in Fig. 2A). All stimulus elements were scaled with eccentricity, as detailed previously (Norcia et al.^33^). Stimuli were presented on a SONY PVM-2541 monitor (1920 × 1080 pixels) and viewed monocularly at a distance of 70 cm. Display pipeline delays and EEG pipeline delays were measured with a photocell and have been corrected for. All probes in the array flickered at 2.727 Hz synchronously with an identical temporal waveform. SSVEPs were measured using probes of 20% contrast, with contrast defined using the Weber definition: C=L probe -L pedestal L pedestal , where *L* is the luminance of the probe or the pedestal.

### Procedure

Participants viewed ON- and OFF-pathway biasing stimuli monocularly, with the contralateral eye covered with an opaque patch. The trials were blocked by eye. Within an eye-testing block, increment and decrement trials were presented in a random order. Trials lasted up to 13.2 seconds with 3000 + 500-ms intertrial intervals. Participants were instructed to withhold blinking and to fixate on the center element. For the conditions of present interest, participants saw 10 trials per condition, per eye (i.e., 40 trials total 2 pathways × 2 eyes × 10 trials). To control participant vigilance and clamp attention at a more constant value, a simple fixation task was presented concurrently during steady-state stimulation. Note, the conditions being analyzed here are a subset of 12 total conditions the participants saw during a session. The other conditions were designed to optimize/examine unrelated stimulus parameters.

### EEG Recording and Artifact Rejection

The EEG was recorded over 128 channels using Hydrocell SensorNets and NetStation 5.2 software (Electrical Geodesics, Eugene, OR, USA). Prior to recording, individual electrodes were adjusted so that the impedance values were lower than 100 kΩ. The raw EEG was amplified (gain = 1000 at 24-bit resolution) and digitally filtered with a 0.3- to 50-Hz bandpass filter. The data were then processed using in-lab software written in Objective C as follows. First, consistently noisy individual channels were detected, rejected, and substituted with the average of the six nearest-neighbor channels. Channels were classified as consistently noisy if over 15% of samples exceeded 30 µV (excluding breaks). After this, the data were rereferenced to the common average. Second, the 13.2-second trials were binned into 12 bins of 1.1 seconds. The first and last bins of each trial were always discarded. Third, to reject data containing coordinated muscle movements and blinks, 1.1-second bins were excluded for all channels if more than 5% of channels exceeded an amplitude threshold of 60 µV. Fourth, 1.1-second bins of individual channels were excluded if more than 10% of samples exceeded 30 µV. These light-touch artifact rejection criteria were derived empirically for adults over hundreds of previous recordings. They readily pick out muscle and blink artifacts as well as electrode motion artifacts, which are not of neural origin, leaving relatively clean EEG. As one of our objectives was to realistically examine the clinical diagnostic potential of the SSVEP, we initially opted not to reject patients’ or controls’ data wholesale on the basis of signal strength or data quality metrics. However, we also present additional classification where low-SNR eyes were rejected (see Supplementary S1 for details on rejection methodology).

### Spectral Analysis

Spectral analysis was performed for each participant, for every sensor, at every 1.1-second bin using a recursive least squares (RLS) filter (Tang & Norcia^34^). Briefly, RLS is equivalent to the discrete Fourier transform (DFT) but is more effective when short trial lengths are used. Conceptually, RLS directly fits sine and cosine waves at selected stimulus-relevant frequencies to a time series. This process yields complex-valued estimates of harmonic amplitudes that can be used in the same way as the amplitudes from the DFT. In the present work, RLS was performed up to the fourth harmonic of the stimulus frequency. Assuming a participant had no bins rejected, this yielded 80 spectral estimates per condition, per eye (10 bins × 10 trials × 4 harmonics). Individual participant trials and bins were averaged within each eye/contrast polarity condition to generate mean sine and cosine coefficients for the first four harmonics of stimulus frequency, 1F, 2F, 3F, and 4F, where F = 2.73 Hz. Note that we use an “xF” nomenclature when referring to stimulus-related frequencies, such that “2F” refers to the second harmonic of the stimulus frequency.

### Spatial Filtering

Group-level reliable components analysis (RCA) in the frequency domain was used to reduce the dimensionality of the 128-channel data to a smaller number of more easily interpreted components, as previously detailed.^24^ Briefly, each reliable component (RC) is a weighted sum of electrical potentials across all channels. The weight vectors are derived through an eigenvalue decomposition performed on a 128 × 128 matrix, where each element represents the ratio of within-trial covariance (*R_xx_*) to cross-trial covariance (*R_xy_*). Solving this decomposition provides multiple ranked components (i.e., spatial filters) that maximize *R_xx_*/*R_xy_*, with the first RC containing the maximal contribution from channels with consistent cross-trial activity. This reflects a fundamental quality of the SSVEP, where repeated presentations of the same stimulus produce similar stimulus-locked neural activation across multiple trials. For classification, RCA spatial filters were trained at the group level for the control participants. Components were learned from the complex values of the first four harmonics of the stimulus frequency, as higher harmonics had a low SNR, even in control eyes.^18^^,^^33^ The raw data for both patients and controls were then projected through the control-derived weightings. We projected all participants’ data through the same weights to eliminate the possibility of differentiating the groups on the basis of a component sign-flip (due to the nature of eigenvalue decomposition). We consider the first six RCs for binary classification. The individual subject-level data comprised their cross-trial vector mean for each harmonic and condition. Finally, individual complex-valued vector means were transformed into amplitude (ρ) and phase delay (θ) values. Phase delay is a circular variable, meaning it cannot be used directly for binary classification. So, we transformed it into the cos(θ) and sin(θ). This process yields the useful property of separating features into amplitude- and phase-derived components. We retained six RCA components and thus had 144 response features per eye (i.e., [cos(θ), sin(θ), ρ] over 4 harmonics × 6 RC components × 2 pathways).

### Case-Control Classification

To evaluate the diagnostic value of the ON- and OFF-pathway SSVEP for glaucoma, we trained a logistic regression classifier with lasso and elastic-net regularization on our dataset of 169 individuals using the glmnet package (V4.18).^35^ We used 10-fold cross-validation (CV) to optimize the regularization parameters of the model and to produce robust performance metrics for both classification algorithms. Each individual was classified as a patient or a control. We trained classifiers using 144 VEP response features and two demographic features (sex and age). We employed two CV strategies for logistic regression. In the first strategy, observations belonging to a given participant were always grouped together in the same CV fold, preserving the dependency structure and preventing overly optimistic performance estimates. We refer to this as the “paired eyes” strategy. For the second strategy, observations were randomly assigned to CV folds, such that the aforementioned dependency structure was not preserved. We refer to this as the “unpaired eyes” strategy. We report classification performance on the basis of accuracy, sensitivity, specificity, and AUC but focus on AUC for model comparisons. For each error metric, we report the CV error, which is the recognized best practice for estimating the prediction error of a model.^36^^–^^38^

Statistical comparisons between AUCs of different classifiers were performed using DeLong's test,^39^ and the differential number of features retained between the ON and OFF pathways was tested with a χ^2^ test. All tests have been corrected for multiple comparisons using the Bonferroni correction. Tests have been grouped according to the hypothesis they are testing and corrected within this group.^40^ The main hypotheses tested are as follows: (1) lack of consideration for paired eyes inflates model performance (3 tests), (2) the ON and OFF pathways have different classification performance (10 tests), (3) phase and amplitude features have different classification performance (10 tests), and (4) removing low-SNR eyes changes performance (3 tests).

## Results

**Figure 3.**
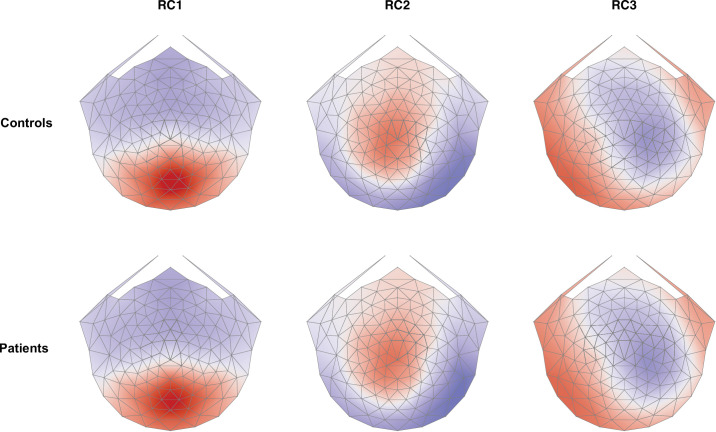
Scalp topography of the first three RCs for controls. The color maps are on the same arbitrary scale. For each topography, north is toward the nasion, and south is toward the inion.

We begin with a brief overview of the data used for classification. Figure 3 shows the scalp topography of the first three RCs for controls. The focal distribution of RC1 over posterior channels is consistent with responses originating from early cortical processing nodes, like primary visual cortex (V1), V2, and V3 (see the forward modeling work of Ales et al.^41^). Reliable components 2 and 3 have more lateralized topographies, likely reflecting downstream visual processing.

### Ignoring Dependency Inflates Performance

Individuals' left and right eyes, as measured by MD and VEP responses, are correlated (see Fig. 1). If one wishes to use both eyes in a classification analysis, it is important to account for this dependency structure to restrain overly optimistic classification performance.^42^ We have accomplished this by keeping the eyes belonging to an individual in the same fold in k-fold cross-validation. The effect of respecting this dependency structure is demonstrated in Table 1, where we report the classification performance metrics. This table contains the rates for six classifiers in total (3 pathway combinations × 2 CV techniques). First, we draw attention to the rows falling under the “paired CV” and “ignore-pairing CV.” Here, classifiers that ignored pairing falsely outperformed those that considered pairing; the increase in AUC across all three classifier columns averaged approximately 4%. In all forthcoming sections, we compare the AUCs of different classification strategies using DeLong’s test (see the Methods section for details) and report the difference in AUC (“Δ AUC”). The AUC increase for pairing ignored was significant when considering both the ON and OFF pathways for classification (Δ AUC = 0.05; 95% confidence interval [CI], 0.03–0.08; *P* < 0.001), just the OFF features (Δ AUC = 0.03; 95% CI, 0.01–0.05; *P* < 0.05), and just the ON features (Δ AUC = 0.03; 95% CI, 0.01–0.06; *P* < 0.05). Note that this spuriously better performance in the pairing-ignored case is because, in a given CV fold, the training dataset is not independent from the test dataset due to some participants having eyes (which are correlated) in both datasets at once. Overall, paired CV classification performed moderately, with AUCs of 0.69, on average. For the remainder of the Results section, we will focus on reporting paired-eye analyses.

**Table 1.** Paired versus Unpaired Classification Metrics for ON and OFF Features

	Both	ON	OFF
Paired CV
Accuracy	0.685	0.637	0.678
Sensitivity	0.768	0.689	0.797
Specificity	0.579	0.571	0.529
AUC	0.708	0.671	0.696
Ignore Pairing CV
Accuracy	0.719	0.656	0.703
Sensitivity	0.802	0.751	0.808
Specificity	0.614	0.536	0.571
AUC	0.762	0.681	0.725

### Similar ON- and OFF-Feature Performance Overall

**Figure 4.**
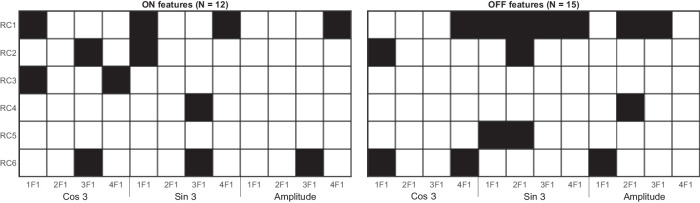
Plot of feature selection matrix after training using paired-eye CV. Separate plots are provided for ON-pathway (*left*) and OFF-pathway (*right*) pathway features. *Dark cells* indicate retained features. Fifteen OFF-related features are retained compared to 12 ON-related features.

From a mechanistic perspective, it is useful to know which features contribute most to classification performance. The AUCs for the paired-eye analysis in Table 1 indicate that classification performance was best when considering OFF-pathway features only or both ON- and OFF-pathway features simultaneously. Classification on the basis of ON-pathway features alone returns a small reduction in performance of ∼0.04 AUC relative to OFF-pathway features, but this difference was not statistically significant (Δ AUC = 0.04; 95% CI, −0.04 to 0.09; *P* = 0.24). To inspect the relative importance of individual features, we report the SSVEP features retained by the elastic-net lasso and their standardized coefficients. We focus on the top 15 features when inspecting feature importance and only look at the performance when both pathways are considered for classification. The matrices in Figure 4 show the features that were retained by the elastic net (with dark cells indicating retained features). The left-most plot is for ON-pathway features and the right-most for OFF-pathway features. Within each plot, RCs are represented on the y-axis, while phase (Cos and Sin of angle), amplitude, and harmonic are represented on the x-axis. First, the OFF-pathway features retained (15) outnumbered the ON-pathway features retained (12)—a small difference that is nonsignificant according to a χ² test (χ²(1) = 0.182, *P* = 0.67). Qualitatively, the ON-pathway features are scattered among the matrix, while the OFF-pathway features appear more concentrated in RC1, particularly in the phase-related components. This phase specificity is consistent with the notion that the phase of the OFF pathway is affected by glaucoma,^18^ though here this is not sufficient to significantly improve classification when using only OFF pathway–related features.

**Figure 5.**
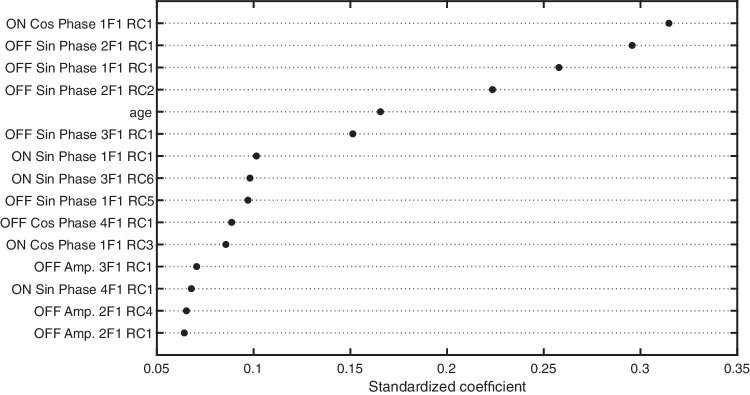
Standardized coefficients for the top 15 features from the GLMNET classification.

To provide a deeper insight into the features that are driving classification performance, it is useful to inspect the ranked feature coefficients returned by the logistic regression. In Figure 5, we inspect the standardized model coefficients for the top 15 retained features. First, the top five SSVEP features are all phase-related features, and the feature with the highest coefficient is an ON-related feature. Overall, in Figure 5, there is a clear indication that phase-related features are driving performance. Also, it is noteworthy that age was a retained feature and that it has the fifth-highest standardized coefficient. This does not mean that age was an exceptionally useful feature. Rather, this suggests that many of the SSVEP features are making a small individual contribution to classification performance. However, there are many SSVEP features, and they outperform age as an ensemble. As a supplementary analysis, we modeled a logistic regression on age alone, finding that its performance was near chance with an AUC of 0.55. Comparing the AUCs between the age-alone model and an age-plus-SSVEP model (with the latter using both ON and OFF features), we find a significant increase in classification performance with the addition of SSVEPs over age alone (Δ AUC = 0.16, *P* < 0.001; 95% CI, 0.07–0.25). Because age is a relatively poor predictor, we will disregard it moving forward. Finally, it is notable that several of the features with the highest model coefficients are at the second harmonic frequency (2F1), not the fundamental frequency. Indeed, a unique aspect of our work is the consideration of multiple response harmonics for classification, and it is evident that they are contributing to classification performance.

### Response Phase Contributes Significantly to Classification

Existing work classifying glaucoma on the basis of the isolated check VEP focuses on signal amplitude–derived features, but there is evidence that the response phase is also affected.^18^ Consistent with this notion, most of the features with the highest coefficients are phase related. To examine the contribution of phase features more closely, we reran classification but considered phase and amplitude features separately. These results are shown in Table 2. Here, classification on the basis of phase-only features performs better than classification on the basis of amplitude-only features. The phase/amplitude pairwise comparisons are significant when considering both pathways for classification (Δ AUC = −0.12; 95% CI, −0.20 to −0.05; *P* < 0.05), only ON features (Δ AUC = −0.15; 95% CI, −0.23 to −0.08; *P* < 0.001), but not when considering only OFF features (Δ AUC = −0.08; 95% CI, −0.15 to 0.01; *P* = 0.52). This latter result suggests that OFF-pathway classification may benefit less from the added phase information than the ON-pathway classification. Notably, the best phase-only performance is on par with the best paired-eye classification performance when considering phase and amplitude simultaneously (see Table 1), suggesting that the amplitude features are contributing disproportionately to classification error.

**Table 2.** Classification Metrics for the Amplitude and Phase of ON and OFF Features

	Both	ON	OFF
Amplitude only
Accuracy	0.606	0.599	0.621
Sensitivity	0.802	0.898	0.836
Specificity	0.357	0.221	0.35
AUC	0.589	0.525	0.62
Phase only
Accuracy	0.688	0.653	0.688
Sensitivity	0.746	0.718	0.797
Specificity	0.614	0.571	0.55
AUC	0.712	0.679	0.695

As an additional analysis, we also compared the ON and OFF performance within both phase and amplitude. Again, there is a trend toward an OFF-pathway advantage in all classifiers in Table 2. The ON versus OFF difference is nonsignificant for phase features (Δ AUC = 0.02; 95% CI, −0.05 to 0.08; *P* = 0.85) but is significant and quite large (in favor of OFF-pathway performance) when only considering amplitude features (Δ AUC = 0.095; 95% CI, 0.03 to 0.16; *P* < 00.05). Overall, these results suggest that the inclusion of phase information significantly improves classification performance, especially in ON-pathway features. The ON-pathway performance is near chance (AUC = 0.53) if only amplitude information is available, where OFF pathway classification is significantly better (by ∼0.10 AUC points).

### Removing Low-SNR Eyes Reduces OFF-Amplitude Advantage

It is possible that the better classification in the amplitude domain for the OFF pathway is due to the smaller (and thus more difficult to measure) signals in the ON pathway.^33^ If this is the case, ON and OFF feature-based classification should be similar when the SNR is high. So we sought to investigate how the removal of low-SNR eyes would affect classification performance. An eye could have a low SNR for a variety of reasons, with severe vision loss and/or a low number of valid SSVEP trials being possible explanations. We redid the analysis on a version of the dataset in which low-SNR eyes had been removed (see Supplementary S1 for details of the SNR rejection mask). This mask removed 19 patient eyes and 25 control eyes.

We present the paired-eye results for these masked data in Table 3. Although there is a trend toward an improvement in classification performance across the board, it is not statistically significant based on an unpaired DeLong’s test when classifying on the basis of OFF-pathway features (original AUC = 0.70; 95% CI, 0.64–0.75; masked AUC = 0.75; 95% CI, 0.69–0.80; *P* = 0.74), ON-pathway features (original AUC = 0.67; 95% CI, 0.61–0.73; masked AUC = 0.74; 95% CI, 0.68–0.80; *P* = 0.37), or both (original AUC = 0.71; 95% CI, 0.65–0.77; masked AUC = 0.75; 95% CI, 0.69–0.80; *P* = 1). In Table 4, we compare amplitude and phase-based classification in the masked dataset. Classification performance was not significantly different between the ON- and OFF-pathway features when considering both phase and amplitude (Δ AUC = 0.007; 95% CI, –0.06 to 0.07; *P* = 0.93) or when phase (Δ AUC = 0.03; 95% CI, –0.04 to 0.10; *P* = 0.93) or amplitude (Δ AUC = −0.007; 95% CI, –0.08 to 0.07; *P* = 0.93) was separately considered. This suggests that removing low-SNR eyes reduced the differential performance between the ON and OFF pathways in the amplitude domain, as this difference was significant in the unmasked data. Phase again outperformed amplitude when comparing classifiers trained on features from both pathways (Δ AUC = −0.11; 95% CI, −0.18 to −0.04; *P* < 0.05), only the OFF-pathway features (Δ AUC = −0.15; 95% CI, −0.22 to −0.07; *P* = < 0.001), but not only the ON-pathway features (Δ AUC = −0.11; 95% CI, −0.20 to −0.03; *P* = 0.09) (the latter did not survive correction for multiple comparisons). The absence of a phase/amplitude difference in the ON pathway is difficult to interpret because the mean difference (Δ AUC) is similar to the difference yielded when both the ON and OFF pathways are considered simultaneously. This may suggest that the ON-pathway performance is simply more variable, which is supported by the ON-pathway phase/amplitude difference having the widest confidence interval. With this caveat, phase features generally outperform amplitude features in the SNR masked dataset. Overall, the pattern of results corroborates the importance of phase features and suggests that amplitude features become more useful when the SNR is high.

**Table 3.** Paired Classification Metrics for ON and OFF Features in SNR Masked Data

	Both	ON	OFF
Paired CV
Accuracy	0.707	0.733	0.707
Sensitivity	0.797	0.823	0.816
Specificity	0.583	0.609	0.557
AUC	0.746	0.737	0.745

**Table 4.** Classification Metrics for the Amplitude and Phase of ON and OFF Features in SNR-Masked Data

	Both	ON	OFF
Amplitude only
Accuracy	0.626	0.623	0.652
Sensitivity	0.848	0.873	0.835
Specificity	0.322	0.278	0.4
AUC	0.644	0.621	0.614
Phase only
Accuracy	0.707	0.733	0.707
Sensitivity	0.797	0.823	0.816
Specificity	0.583	0.609	0.557
AUC	0.746	0.737	0.745

## Discussion

Our main finding is that response phase features of the SSVEP to ON- and OFF-pathway biased stimulation contribute more to the success of glaucoma patient classification than do amplitude features for both feature types. In our full dataset, when considering all possible SSVEP features, we find comparable sensitivity for both OFF- and ON-pathway biased SSVEP features under our conditions of measurement. However, classification performance using OFF-pathway amplitude features is significantly better than the same using ON-pathway features. These results concerning amplitude features are compatible with our recent electrophysiological work, indicating that the OFF pathway is preferentially affected by glaucoma.^18^ However, it is evident that when considering both phase and amplitude, using OFF-pathway features alone does not meaningfully improve glaucoma classification rates, and the absence of a gross improvement in classification performance for OFF-pathway features is replicated in an SNR-masked version of the dataset. Considering the ON feature phase is sufficient to narrow the gap between ON and OFF feature performance based solely on amplitude. Our findings are discussed below in the context of previous work.

### Relative Utility of OFF- and ON-Pathway Response Features

The early icVEP small-sample findings suggested that the neural response to ON-pathway biasing stimuli is more useful than responses to OFF-biasing stimuli for glaucoma classification.^43^ Zemon and colleagues,^43^ using 10-Hz sinusoidal stimulation at 15% contrast, found that AUCs were higher for incremental stimuli than decremental stimuli (0.94 vs. 0.82). At 10% contrast, they found the opposite: AUCs were higher for decrements than increments (0.91 vs. 0.73%, respectively). This ∼0.04 best AUC difference between ON- and OFF-biasing stimuli (which is similar to the trend OFF advantage we report in the whole-dataset analysis) formed the published basis of subsequent work that only considers responses to ON-biasing stimuli.^2^^–^^10^ The stimuli we used were at an intermediate contrast of 20%, but our results suggest that the differential ON and OFF feature contribution to classification is small. If the relative utility of ON and OFF features depends on the contrast being tested, it is plausible that our use of an intermediate (20%) contrast stimulus (as opposed to 10% or 40%) brought the informational value of ON- and OFF-pathway features closer together. In our data, the only significant indication of greater OFF-pathway performance was restricted to the amplitude features in the nonmasked data. That this finding is absent in the masked data could suggest that the ON-pathway classification is more vulnerable to floor effects from poor signal quality^43^ due to having a generally lower SSVEP amplitude.^33^ Indeed, it may be desirable to use OFF-biasing stimuli solely because they produce larger signals, particularly because low signal can be due to confounds unrelated to eye disease such as inattention and difficulty fixating.

### Response Latency Is Useful for Glaucoma Classification

Abnormal VEP latencies in glaucomatous eyes have been reported previously in human^26^^–^^30^ and macaque^25^ studies that do not use signed-contrast stimuli. There is also evidence that glaucoma leads to optic nerve demyelination,^31^^,^^32^ which may increase transduction delay. Using low-frequency sign-reversing checkerboard stimuli, Zhong et al.^28^ and Parisi et al.^30^ reported that the P100 latency was increased by approximately 30 ms in glaucomatous eyes. Using ROC analysis, Parisi et al.^30^ reported perfect case-control classification accuracy based on P100 latency (better than P100 amplitude alone) in their cohort of 84 patients with open-angle glaucoma. Using similar stimuli, Pillai et al.^27^ found response latency to classify primary open-angle glaucoma from controls well when using high contrast stimuli (AUCs of 0.97) and low contrast stimuli (AUCs of 0.86), both better than classification on the basis of amplitude (AUCs of ∼0.74). More recently, using the positive check icVEP and pattern VEP (pVEP), Wang et al.^9^ found that the latency and amplitude components of the pVEP P100 had comparable classification performance (AUCs of ∼0.80). While both P100 components were inferior to the icVEP SNR AUCs (∼0.89), the icVEP response is likely subject to the same signal transduction mechanisms presumably affected by glaucoma, suggesting that the phase component of the icVEP should be considered in future work. Dunn and colleagues^25^ measured electroretinograms in macaques with induced glaucoma, with the only indication of worse OFF-pathway function being in the implicit time of the d-wave. While we did not find the OFF-pathway phase to significantly outperform the ON-pathway phase (which could be due to a number of methodological and animal model differences), the work by Dunn et al.^25^ supports the notion that response latency carries information about glaucoma. Importantly, the SSVEP work of Norcia et al.^18^ demonstrated that a change in the difference in phase between the ON and OFF pathways is apparent in early glaucoma, even when response amplitudes are indistinguishable from controls. The potential diagnostic value added by phase features in the icVEP should be investigated in future work.

### Moderate SSVEP Classification Performance

Despite our novel inclusion of both eyes in training and having many more VEP features than previous work, our overall classification rates are lower than those reported in the icVEP literature. In our paired-eyes analysis of the full dataset, our best AUC was ∼0.71%, and the best accuracy obtained was 69% (for both ON and OFF features in the logistic regression). For comparison, AUCs of 0.77 to 0.94 have been reported in the icVEP literature using incremental sinusoidal modulation and a simple single-harmonic SNR criterion.^2^^,^^4^^,^^5^^,^^7^ Why are our AUCs so comparatively low? First, it should be noted that we used 10-fold cross-validation to produce robust performance estimates and minimize overfitting. That is, the estimates of model performance we have calculated are derived from out-of-fold data that the model has not seen. This restrains overly optimistic classification performance, resulting in less accurate but likely more generalizable predictions.^44^ Indeed, the absence of cross-validation in existing icVEP work may limit the interpretation of those results. That is, it is possible that the ROC curves obtained are specific to a given dataset due to variations in site-specific factors and differences between patient groups. These variations likely contribute to the variety of best SNR thresholds and AUCs reported in the icVEP literature. Using some form of train-test splitting (as we have done with cross-validation) to verify that the metrics obtained are robust (at least within a dataset) is recommended. Additionally, while we have controlled for correlation between paired eyes by allocating participants’ eyes to the same cross-validation fold, it is possible that electrophysiological differences between eyes could also be predictive of disease. Information from both eyes is clinically available and, when used appropriately, could further improve disease detection.

If we ignore the details of model assessment, the classification performance we obtain is likely insufficient for direct clinical use. Classification performance may benefit from a more detailed exploration of how glaucoma affects the VEP contrast response function of each pathway, which would inform the best contrasts to choose for diagnostic purposes. We have used a single stimulus contrast, and it is possible that the parameters of our current experimental stimuli produce a VEP that is less discriminable between the patient and control groups. For example, Xu et al.^45^ examined icVEP classification performance at several ON-biasing contrasts. They found that classification performance deteriorated with increasing contrast, with 8% and 14% contrast checks yielding AUCs of 0.76 to 0.78, while 22% contrast checks reduced to 0.69. Notably, one of their worst-performing stimulus contrasts is close to the one we used here and reports an AUC similar to that of our whole-dataset analysis. Interestingly, this higher-contrast condition would have evoked a stronger visual response, suggesting that their results are not limited by an SNR floor at low contrasts (counter to the floor effect previously proposed^43^) and that a pathological alteration to low-contrast gain may produce a clearer separation between patients with glaucoma and controls. Investigating the effect of glaucoma on the shape of the ON- and OFF-pathway contrast response function would likely be useful for improving classification performance, as there may be a contrast at which disease detection is optimal. If this is the case, it may explain why ON- and OFF-pathway classification performance is similar in our dataset, which is not the case when different contrast levels are used.^43^

Another possibility is that our overall classification performance was limited by the data we collected, as we conducted relatively few trials in the ON- and OFF-biasing conditions of our experiment. This is because these two conditions were part of a collection of 12 conditions used to optimize other stimulus parameters, meaning we obtained only up to ∼100s of VEP data for each condition. The removal of low-SNR participants markedly improved ON-pathway amplitude-based classification, suggesting that overall performance would benefit from more trials per participant. This may also be alleviated by a multifrequency stimulus, where the upper and lower visual fields modulate at a different frequency, as upper and lower visual field signal cancellation (due to V1 morphology) would be eliminated. Additionally, we did not collect eye-tracking data to ensure fixation was maintained for the duration of a trial, and many participants had trials that were rejected on the basis of eye blinks and muscle EEG artifacts. Existing icVEP glaucoma classification work using commercially available icVEP acquisition systems continuously monitors gaze to ensure a specified amount of high-quality data is collected. Ensuring that a full set of high-quality trials is collected for every participant allows the use of statistical normalization methods that have been shown to improve icVEP classification performance.^4^ Indeed, it is possible that the improved classification we find for phase over amplitude will be lessened if robust signal normalization were performed, as uninformative between-subject variability would be reduced. However, the requisite fine-grain real-time monitoring was not possible at the time of data acquisition using our equipment.

## Conclusions

The present results suggest that the visual response phase contains useful information for the classification of glaucoma. Classification on the basis of OFF-pathway amplitude may be more robust to low SNR than ON-pathway amplitude, but when considering both amplitude and phase, the differential performance of these pathways is similar. Given the numerous other differences between our approach and those used in the isolated check literature, future studies would do well to continue to perform direct comparisons of results from different forms of ON- versus OFF-pathway selective stimulation at different contrasts. Perhaps more importantly, future SSVEP work should consider adding phase-related features at multiple response harmonics (where possible) as their inclusion may produce better-performing classifiers.

## Supplementary Material

Supplementary S1
