# Supplementary material for: Glaucoma Classification Through SSVEP-Derived ON- and OFF-Pathway Features
Source: Transl Vis Sci Technol. 2026 Jan 5;15(1):2. doi: 10.1167/tvst.15.1.2 (PMC12782199; doi:10.1167/tvst.15.1.2)
Supplement: Supplementary S1 [file tvst-15-1-2_s001.pdf]

Supplement 1: Exclusion of low SNR eyes.

To remove low signal-to-noise eyes from the dataset, the following was applied to every patient and every control. The SSVEP data for incremental and decremental visual stimulation for both the left and right eyes were loaded into MATLAB. Note, we consider an eye to be the minimum rejectable unit of data (i.e., we won't reject the ON-biased only from a single eye or both eyes). For each eye, across both the ON- and OFF- biased responses, we generate an individual-level reliable components analysis[1] (RCA) filter at stimulus relevant frequencies. We reason that this RCA filtered data captures most of the stimulus locked activity for the participant, and project their raw data through this filter for further analysis. The RCA filtered data for the first two harmonics of both pathways in both eyes are submitted to a t-circ[2] statistical test (eight tests in total for each participant). The p-values of these tests are then adjusted for multiple comparisons on a per-eye basis using the Benjamini-Hochberg procedure.[3] We used a generous false discovery rate of 0.25, reasoning that we did not want to reject too many individuals who may have a genuine visually evoked potential. After this correction, if an eye possessed at least one statistically significant test for both the ON and OFF pathways, it was kept in the data. All other cases were rejected.

1. Dmochowski, Jacek P., Alex S. Greaves, and Anthony M. Norcia. 2015. "Maximally Reliable Spatial Filtering of Steady State Visual Evoked Potentials." *NeuroImage* 109 (April): 63–72.
2. Victor, J. D., and J. Mast. 1991. "A New Statistic for Steady-State Evoked Potentials." *Electroencephalography and Clinical Neurophysiology* 78 (5): 378–88.
3. Benjamini, Y., and Y. Hochberg. 1995. "Controlling the False Discovery Rate: A Practical and Powerful Approach to Multiple Testing." *Journal of the Royal Statistical Society Series B-Methodological* 57 (1): 289–300.
